# Transcription networks responsible for early regulation of *Salmonella*-induced inflammation in the jejunum of pigs

**DOI:** 10.1186/1476-9255-10-18

**Published:** 2013-04-17

**Authors:** Marcel Hulst, Mari Smits, Stéphanie Vastenhouw, Agnes de Wit, Theo Niewold, Jan van der Meulen

**Affiliations:** 1 Livestock Research of Wageningen University and Research Centre, P.O. Box 65, Lelystad, 8200AB, The Netherlands; 2 Central Veterinary Institute of Wageningen University and Research Centre, P.O. Box 65, Lelystad, 8200 AB, The Netherlands; 3 Present address: Nutrition and Health, Katholieke Universiteit Leuven, Kasteelpark Arenberg 30, Heverlee, B, 3001, Belgium

**Keywords:** Transcription regulation, *Salmonella*-induced inflammation, Pig intestine

## Abstract

**Background:**

The aim of this study was to identify transcription factors/regulators that play a crucial role in steering the (innate) immune response shortly (within a few hours) after the first contact of the intestinal mucosa with an inflammatory mediator, and to test whether the processes regulated by these factors/regulators can be modulated by chemical substances of natural origin.

**Methods:**

We experimentally induced inflammation by perfusion of surgically applied jejunal loops with *Salmonella enterica* subspecies *enterica* serovar Typhimurium DT104 in three pigs. Segments of mock and *Salmonella* treated loops were dissected after 2, 4 and 8 hours of perfusion. IL8 and IL1-beta mRNA expression levels were measured in mucosal scrapings of all segments. Furthermore, intra-animal microarray comparisons (isogenic) between *Salmonella* and mock treated segments after 8 hours, and inter-animal comparisons between similar *Salmonella*-treated loops of each pig at 2 and 4 hours, were performed.

**Results:**

IL-1beta and IL8 mRNA levels, and intra-animal microarray comparisons at 8 hours between *Salmonella* and mock treated segments showed that the response-time and type of response to *Salmonella* was different in all three pigs. This plasticity allowed us to extract a comprehensive set of differentially expressed genes from inter-animal comparisons at 2 and 4 hours. Pathway analysis indicated that many of these genes play a role in induction and/or tempering the inflammatory response in the intestine. Among them a set of transcription factors/regulators known to be involved in regulation of inflammation, but also factors/regulators for which involvement was not expected. Nine out of twenty compounds of natural origin, which according to literature had the potential to modulate the activity of these factors/regulators, were able to stimulate or inhibit a *Salmonella*-induced mRNA response of inflammatory-reporter genes IL8 and/or nuclear factor of kappa light polypeptide gene enhancer in B-cells inhibitor alpha in cultured intestinal porcine epithelial cells.

**Conclusions:**

We describe a set of transcription factors/regulators possibly involved in regulation of “very early” immune mechanism which determines the inflammatory status of the intestine later on. In addition, we show that these mechanisms may be modulated by chemical substances of natural origin.

## Introduction

Multiple immune cells are involved to sense “danger signals” and activate and control a local immune response in the mucosa of the gastrointestinal (GI) tract. Resident and infiltrating immune cells collaborate with functional epithelial cells to respond to pathogens and toxic residues formed after digestion of feed/foods. Specialized cells (e.g. M cells) and enterocyte-conditioned dendritic cells (DC’s) imbedded in the epithelial layer of the intestine constantly survey the luminal environment for antigens and activate innate as well as adaptive defense mechanisms [1,2]. Derailment of this system often results in excessive inflammatory reactions. In case this state of inflammation lasts to long serious damage is imposed to the mucosal layer resulting in loss of its barrier function. Intestinal diseases like Crohn’s disease and Ulcerative colitis (inflammatory bowel diseases) are characterized by a chronic state of inflammation of the small and/or large intestine causing abdominal pain and diarrhea, and consequently, to ongoing mal-absorption of nutrients [3]. To suppress excessive inflammation patients are treated with drugs mostly in combination with specific diets. At first harmless symptomatic drugs are used. In case these drugs do not reduce symptoms, inflammation reducing drugs are prescribed. However, long-term use of this later group of drugs can provoke serious side effects [4]. In farm animals, inflammation of the GI tract, e.g. provoked by hostile pathogens during weaning, also causes mal-absorption, and consequently, growth delay. However, in the last decade prophylactic and therapeutic use of growth-promoting antibiotics in farm animals in order to reduce economic losses is controversial because of the risk of releasing antibiotic resistance in the environment. Therefore, in the last two decades the development and use of alternative ‘natural’ products” like food/feed additives and probiotic bacteria to activate or temper immunological reactions in the GI tract have gained more attention. Also the awareness of consumers about the principle “what you eat affects your health” has increased tremendously, and with this consumption of “health improving” food products. However, the knowledge about the mechanisms how these additives and probiotics do their job in a complex environment as the GI tract, is still limited [5]. This hampers the development and application of more effective and cheap natural additives to improve intestinal health in both humans and animals.

The aim of this study was to identify networks of transcription factors/regulators that play a crucial role in steering the (innate) immune response shortly after the first contact (within a few hours) of the intestinal mucosa with an inflammatory mediator. Based on these networks chemical substances of natural origin can be selected which have the potential to influence the activity of such transcription factors/regulators, and with this, the inflammatory state of the intestine.

The bacteria *Salmonella enterica* subspecies *enterica* serovar Typhimurium DT104 (hereafter denoted as *Salmonella*) is an important disease in animals and humans. It infects cells lined up in the epithelial layer of the small and large intestine and may cross this barrier to invade the lamina propia and to produce a systemic infection [6]. Interaction of *Salmonella* with epithelial cells of the intestinal mucosa induces pro-inflammatory responses characterized by the release of several cytokines and chemokine’s [7]. Earlier, we showed that IL8 mRNA expression by enterocytes was triggered rapidly (4–8 hours) after encountering pathogenic bacteria like *Salmonella* and ETEC, or toxins produced by these bacteria [8,9]. Furthermore, in cultivated porcine epithelial cells (IPEC-J2) infected with *Salmonella* also an enhanced expression of IL8 was observed [10]. Together with the capability of these cells to express several other cytokines (IL1A , IL6, IL7, IL18, TNFA and GMCSF), this inducible IL8 expression makes IPEC-J2 cells a valuable *in vitro* model to study the contribution of enterocytes in the regulation of immune mechanisms in the intestine [10].

Recently we studied the transcriptional response of intact intestinal mucosa after infection with *Salmonella* in our *in situ* Small Intestinal Segment Perfusion model (SISP) [9]. In this experiment, by surgery applied mid-jejunal loops were challenged with and without *Salmonella*. After a relative short period of perfusion (2, 4 or 8 hours) parts of these loops were dissected, allowing comparative time-dependent measurements within one animal (isogenic). Because in this previous study mRNA levels in these loops were measured using a home-made cDNA platform containing a limited number of probes [11] we re-analyzed these samples in this study using a commercial pig oligonucleotide array platform. This commercial platform contained a more global array of probes, enabling us to generate a comprehensive overview of the processes/pathways induced shortly after *Salmonella* exposure. Moreover, the plasticity in time and type of response between individual pigs allowed us to extract a set of genes possibly involved in the transcriptional regulation of inflammation in the jejunum. Based on bioinformatics analysis, chemical substances of natural origin were selected. To assess whether these substances have potential to modulate a *Salmonella*-induced response in enterocytes, we tested their effect on a Salmonella induced IL8 and NFKBIA mRNA response in IPEC-J2 cells.

## Methods

### Small intestinal segment perfusion with *Salmonella*

**Figure 1:**
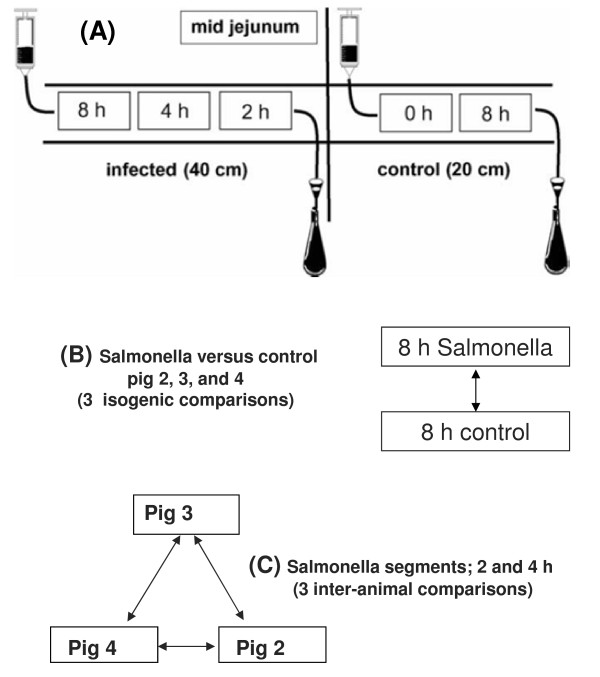
**Design of the “Small Intestinal Segment Perfusion” (SISP) experiment.** (**A**) Surgically applied jejunal loops were perfused without (control) or with Salmonella (infected) and segments were dissected after the indicated hours (0, 2, 4, or 8) [9]. For all three pigs’ (2-4) treatments of intestinal loops were identical. (**B**) Isogenic microarray comparisons between segments dissected from infected and control loops after 8 h of perfusion. (**C**) Interanimal microarray comparisons of Salmonella perfused loops. Segments were dissected after 2 and 4 hours, and at both these time-points 3 interanimal microarray comparisons were performed.

Total RNA isolated from mucosal scrapings of an earlier described SISP experiment was used for micro array and QRT-PCR analysis. In the mid jejunum, intestinal segments were prepared in 4 male piglets (all from the same liter) as described [8]. 10 cm of the control segment (0 hour control) was dissected before segments were perfused for 1 hour without and with *Salmonella enterica* subspecies *enterica* serovar Typhimurium DT104 10^9^ CFU/ml according to the scheme depicted in Figure 1A. Subsequently, loops were perfused for 1, 3 or 7 hours without *Salmonella* and samples were dissected at 2, 4 and 8 hours after the first exposure with *Salmonella* (at the start of the 1 hour perfusion period with *Salmonella*). Details of this SISP experiment, dissection of mucosal scrapings, and RNA isolation from these scrapings was published previous [9]. RNA from mucosal scrapings of three of these four pigs (pig 2, 3, and 4) was stored as alcohol precipitate at −20°C (not sufficient material was available anymore of pig 1). After centrifugation the RNA pellet was dissolved in RNAse-free water, and the integrity of this RNA was checked by analyzing 0.5 μg on a 1% (w/v) agarose gel. The SISP experiment described in Niewold *et. al.* (2007) was approved by the Animal Ethics Commission in Lelystad, the Netherlands, in accordance with the Dutch law on animal experimentation [9].

### Microarray analysis

The commercially printed Pig “Operon” expression micro-array was used for all hybridizations. Array slides contained a total of 13297 70-mer oligonucleotide sequences representing 10655 *Sus scrofa* sequences with a blastn hit to known human, mouse or pig mRNA sequences and some 3’ expressed sequence tags (Operon Array-Ready Oligo Sets™ for the Pig Genome, Version 1.0, plus the Pig Genome Oligo Extension Set, Version 1.0). All probes were printed in duplicate. Dual labeling of total RNA using the RNA MICROMAX TSA labeling and detection kit (Perkin-Elmer), hybridization and washing of slides was performed as described recently, except that 4 μg of template was used instead of 1 μg [8,9]. A total of 6 comparative hybridizations were performed according to the scheme depicted in Figure 1B and C. For each comparison a dye-swap (duplicate) was performed. Slides were scanned and images were gridded on a GenePix 4200A 01 Autoloader 116826 (Molecular Devices, Apeldoorn, The Netherlands). Data files were processed in GenePix Pro 6.1.0.4 or 6.0.1.25 (Molecular Devices, Apeldoorn, the Netherlands). Data normalization (blank-specific background correction, LOWESS fit function with a fraction of 0.2) was performed using a customized version of the statistical software package R for simultaneous data analysis of dye-swaps. Significantly differential expressed probes with M value (Log 2 scale) of < −1.58 or >1.58 (a ratio greater than 3-fold) and with a p-value <0.025 were selected. For each probe 4 spots were hybridized, 2 on one slide and 2 on the dye-swap slide. Probes with more than one missing values were removed from gene-lists used for bioinformatics analysis. Results of these micro array comparisons are posted in the NCBI GEO database (accession number GSE41630)

### Bioinformatics and functional analysis

Oligonucleotide sequences of differential expressed probes not annotated yet, or annotated as Unigene, tentative consensus sequences (TC) or mRNA accession number, were compared with the NCBI non-redundant nucleotide databases using blastn and blastx options to assign a gene-name to these probes. Probes that did not produce a significant match with any other eukaryotic mRNA/gene were excluded from gene lists used for functional analysis. Throughout this manuscript official human gene-symbols (HUGO Gene Nomenclature Committee) were used in the text and in all (supplementary) figures and tables. Response genes detected in inter-animal comparisons were assigned to a list of genes based on their similarity in response to cytokines IL8 at 2 hours and IL1B at 4 hours (see results section for a detailed explanation). Because of the important role of IL8 and IL1B in inflammatory processes, these lists were named after these cytokines. Lists of gene-symbols were uploaded separately in web-based bioinformatics programs.

The Database for Annotation, Visualization and Integrated Discovery (DAVID version 6.7) website [12] and the “Set Distiller” module of GeneDecks [13] were used to assign genes to a specific pathway. Because far more human genes are annotated, and more information in databases is available for humans than for pigs, the human background was used for this functional analysis. In DAVID pathways (KEGG and Biocarta) with a *p*-value of <0.2 (EASE score) were retrieved. In Genedecks pathways (KEGG, MLPR, CST, GeneGlobe Pathway Central, Invitrogen, and Ingenuity) were retrieved called significant with a *p*-value <0.05 using the Set Distiller algorithm. KEGG pathways retrieved from DAVID were only listed when not called significant by Genedecks, or in case more genes were listed than in Genedecks. In case a pathway was called significant in one group (list) of genes, genes regulated on the same time-point in the “opposite” list, and also part of this pathway, were also retrieved from DAVID and GeneDecks.

From DAVID “Functional Annotation charts” transcription factors or genes involved in regulation of transcription were identified by gene-ontology analysis and uploaded as sub-list in GNCPro (free online software developed and maintained by SABiosciences Inc.) to establish relations between these genes (i.e. to build a network of transcription factors and regulators). Non-interacting genes were omitted from the displayed network.

Functional association between proteins encoded by differential expressed genes, ligands, and enzyme substrates/products linked to these proteins, were established using the (protein)-protein-chemical interaction web tool STITCH2 [14]. Relevant chemicals were added to gene lists and uploaded to in STITCH2 to establish associations. Associations with a confidence score of ≥ 0.4 (medium level) were selected from output files and displayed. In Additional file 1: Table S1 the type and confidence level of each association is listed in a separate sheet STITCH interactions.

### Chemical data-mining

Based on bioinformatics analysis (see above) a set of regulated genes was selected from 8, 4 and 2 hours micro array comparisons. Each individual gene/protein was loaded into STITCH2 to find associations with chemical compounds scoring a high confidence level (>0.7). For each gene/protein chemical compounds were selected meeting the following criteria: i) direct interaction with the protein, ii) present in the same (chemical) pathway, iii) capable to inhibit or activate the function of the protein, iii) involved in direct interference with transcription of the gene in question or relevant (groups of) genes transcribed/regulated by this gene/protein. In addition, for each of the selected genes/proteins-chemical combination relevant literature linked in the Comparative Toxicogenomics Database [15] (http://ctdbase.org/) or in PubMed (NCBI) was examined.

### Quantitative PCR

The relative concentration of NFKBIA, IL1B, IL8, TIMP1, MMP1, and REG3A (alias PAP) mRNA in all RNA samples extracted from mucosal scrapings and isolated from IPEC-J2 monolayers was determined by real-time PCR. The gene-specific primers and specifications for these quantifications are recently described (REG3A [9], NFKBIA, TIMP1 and [16], IL1B and IL8 [17]). RT reactions were performed with Superscript III (Invitrogen) and random hexamer primers (pdN6) according to the manufacturer’s instructions using 250 ng of RNA template. The quantity of 18S rRNA in each RNA sample was determined using the above described RT reactions by real-time PCR [18] and used to normalize NFKBIA, IL1B, IL8, TIMP1, MMP1, and REG3A data. The quantity of 18S ribosomal RNA showed no essential differences among all individual RNA samples extracted from mucosa or from IPEC-J2 monolayers.

### IPEC-J2 in vitro assay

IPEC-J2 cells were seeded in 2 cm^2^ tissue culture wells (M24 plate) and grown for 7 days at 37°C and 5% CO2 using 1:1 DMEM/Ham’s F10 1:1 medium (Gibco-BRL) supplemented with 5% FCS without antibiotics [10]. Confluent monolayer were washed twice with medium without FCS (hereafter denoted as medium) and incubated for 1 hour with this medium. Medium was discarded and a mixture of *Salmonella* bacteria and the chemical dissolved in medium was added. In a pilot experiment the multiplicity of infection (MOI) that did not induce visible (microscopic) damage to the cells (in the absence of chemical) was determined after an exposure times of 6 or 20 hours. Based on this pilot experiment a MOI of 1.0 and 0.1 was used for 6 and 20 hours of incubation, respectively. In a similar pilot experiment the effect of the highest concentration chemical was evaluated after 6 and 20 hours. For most chemicals the morphology of the IPEC-J2 cells was changed after 20 hours, but not after 6 hours. For these chemicals a maximum incubation time of 6 hours was used. After incubation total RNA from cells was extracted using Trizol (Invitrogen) according to the manufacturer’s instructions. RNA was treated with DNase as described [8] and further purified using the QIAamp MinElute Virus Spin Kit (Qiagen Cat no. 57704). The integrity of RNA was checked by analyzing an aliquot of 0.5 μg on a 1% (w/v) agarose gel before it was used as template in QRT-PCR reactions. The effect of all chemicals was tested in duplicate at 3 different concentrations. In each culture plate duplicate control wells containing no *Salmonella* (only with chemical), or containing no chemical (only with *Salmonella*), or without chemical and *Salmonella* (only medium), were incubated for the same period as was done for wells containing mixtures of chemicals and *Salmonella*. In case another solvent was needed (e.g. ethanol) to prepare a stock solution of chemicals, control wells without chemical were incubated with medium containing similar concentrations of solvents as were used for wells incubated with chemicals. The percent stimulation or inhibition in Salmonella and in mock challenged wells was calculated from the relative concentration of IL8 or NFKBIA mRNA (mean; n = 2) containing a specific concentration of chemical divided by the mRNA concentration measured in corresponding wells containing no chemical, and multiplied with 100% (e.g. IL8 in percent; {[IL8]+/ [IL8]-}× 100%).

### Cortisol analysis

For determination of cortisol concentrations in mucosal scrapings the Beckman Coulter ELISA was used (cat.no DSL-10-67100i). To extract cortisol from scrapings, 0.5 g was homogenized in 2 ml of PBS in a tube with screw-cap using a tissue homogenizer. Suspensions were incubated for 2.5 hours at 70°C under constant agitation. After cooling to room temperature 5 ml of ethyl ether was added and the mixture was shaken vigorously for 2 min, centrifuged for 10 min at 3000xg, frozen, and stored for 2 hours at −20°C. The ethyl ether fraction was transferred to a clean tube and the ethyl ether was evaporated under liquid nitrogen. The residue was dissolved in 0.5 ml PBS and the concentration cortisol in the extract was analyzed in the ELISA. A linear correlation (Regression Coefficient = 0.9824) was found between the amount of scrapping (g) used for extraction and the response in the ELISA, showing that the extraction method was reliable. The concentration cortisol (ng/g) in extracts was determined by extrapolation on a standard curve. For each mucosal scraping duplicate extracts were prepared and analyzed. A two-sided Grubbs’ test (p < 0.01) using the mean and standard deviation calculated over all determined values was performed to identify outliers; i.e. values that differed significantly from the population.

## Results

### Individual pigs respond differently to *Salmonella*

**Figure 2:**
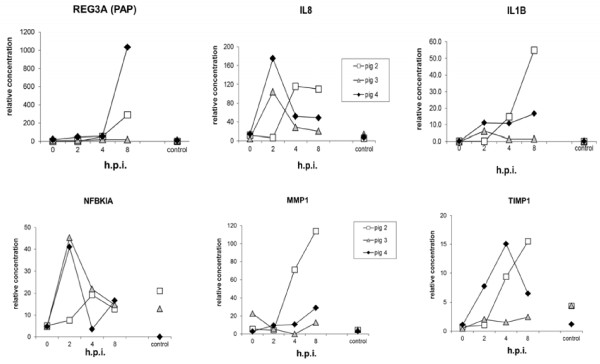
**Relative concentration of REG3A, IL8, IL1B, NFKBIA, MMP1 and TIMP1 mRNA expression measured by QRT-PCR in segments dissected from *Salmonella *treated (2, 4, and 8 h) and mock treated (control; 0 and 8 h) loops.**

In an earlier study a limited number mRNA’s were found differentially expressed at 2, 4 and 8 hours when pools composed of RNA extracted from identical *Salmonella* treated segments of 4 SISP pigs were compared to pools prepared from mock treated loops of these 4 pigs (Figure 1B) [9]. In part the limited number of genes detected was due to the low complexity of the previous used home-made cDNA array. However, quantification of REG3A (alias PAP; a C-type lectin with antibacterial properties) mRNA expression in individual segments of all pigs (see Figure 2) indicated that responsiveness to *Salmonella* differed substantially for individual pigs, and, most likely, also accounted for this. This observed plasticity urged us to analyze IL8 and IL1B mRNA responses by Q-PCR in all segments dissected from SISP pigs 2, 3, and 4 (not sufficient material was available anymore from pig 1). IL8 and IL1B mRNA expression profiles (Figure 2) clearly showed that response-time and the type of response differed for all 3 pigs. In addition, quantification of TIMP1, MMP1, and NFKBIA in all segments confirmed this. The results of these Q-PCR analysis were largely in agreement with the below presented micro array data (see Table 1 and Additional file 1: Table S1).

**Table 1:** Differential expression of genes

**GENE**	**Ratio Salm/Mock 8 h**			**Ratio 2 h**			**Ratio 4 h**			**Description**
	**Pig 2**	**Pig 3**	**Pig 4**	**2/3**	**2/4**	**3/4**	**2/3**	**2/4**	**3/4**	
**# Genes**	**325**	**37**	**164**	**79**	**104**	132	**142**	**49**	**202**	
**NFKBIA (PCR)**	**7.8**	**-**	**5.6**	**0.04**	**0.07**	-	**-**	**-**	**7.2**	Nuclear factor-kappaB inhibitor alpha
**IL8 (PCR)**	**13.1**	**-**	**8.2**	**0.03**	**0.03**	-	**10.7**	**4.1**	**-**	Interleukin-8
**REG3A (PCR)**	**49.8**	**-**	**11.5**	**0.09**	**0.01**	0.03	**14.1**	**-**	**0.07**	Islet of Langerhans regenerating protein 3A (alias; PAP)
**TIMP1 (PCR)**	**13.1**	**-**	**4.7**	**-**	**0.22**	-	**8.2**	**-**	**0.23**	Metalloproteinase inhibitor 1 precursor (TIMP-1)
**IL1B (PCR)**	**37.7**	**3.9**	**47.5**	**0.17**	-	-	**25.9**	**-**	**0.06**	Interleukin-1 beta
**MMP1 (PCR)**	**10.2**	**3.4**	**3.7**	**0.15**	-	-	**13.1**	**-**	**-**	Matrix metalloproteinase-1 (Interstitial collagenase)
**S100A9**	**22.6**	**5.4**	**4.9**	-	-	-	**18.1**	**8.5**	-	S100 calcium binding protein A9 (calgranulin B)
**HSD11B2**	*0.56*	**0.14**	*0.34*	-	-	-	-	**-**	-	Hydroxysteroid 11-beta dehydrogenase 2
**TFF2**	*0.40*	**0.08**	*0.56*	-	-	-	-	**0.13**	-	Trefoil factor 2 (Pancreatic spasmolytic polypeptide)
**HSPA1B**	*1.15*	**10.4**	*0.87*	-	-	-	-	-	-	Heat shock 70 kDa protein 1B
**HSPA1A**	*0.86*	**6.2**	*0.95*	-	-	-	-	-	-	Heat shock 70 kDa protein 1A
**RAC1**	*0.71*	**0.08**	*1.3*	-	-	-	-	-	-	Ras-related C3 botulinum toxin substrate 1
**TNFRSF12A**	*2.1*	**8.5**	*2.7*	-	-	-	-	-	-	TNF receptor superfamily member Fn14
**NR4A1**	*1.9*	**7.2**	**3.0**	-	**0.21**	-	-	-	-	Nuclear receptor subfamily 4, group A, member 1
**IRF1**	**6.4**	**6.4**	**4.1**	-	-	-	-	-	-	Interferon regulatory factor 1
**EGR1**	**4.6**	**5.6**	**4.8**	-	**0.15**	-	**5.8**	**9.7**	-	Early growth response 1
**THBS1**	**14.1**	**9.7**	**4.8**	-	-	-	**8.3**	**-**	**0.15**	Thrombospondin 1
**F3**	*2.0*	**3.1**	**5.3**	**0.24**	-	**-**	**-**	**-**	**-**	Coagulation factor III
**FOS**	**3.3**	**4.2**	**8.0**	**0.20**	**0.17**	**-**	**7.9**	**6.6**	**-**	c-Fos protein
**DMBT1**	**8.9**	**6.9**	**8.2**	-	**0.08**	-	**-**	**-**	**0.09**	Surfactant pulmonary-associated D-binding protein (gp-340)
**PLAUR**	**10.5**	**8.6**	**11.8**	-	-	-	**-**	**-**	**-**	Urokinase plasminogen activator surface receptor
**CYR61**	**11.2**	**18.1**	**36.0**	-	-	-	**-**	**4.9**	**-**	cysteine-rich, angiogenic inducer 61
**IL1RN**	**70.2**	**3.2**	**73.2**	-	-	-	**-**	**-**	**-**	Interleukin-1 receptor antagonist protein

# Genes: Total number of differentially expressed genes with a ratio of 3-fold up- or down-regulated detected in 8 hours isogenic comparisons (*Salmonella* over mock, left panel), and in 2 (middle panel) or 4 (right panel) hours inter-animal comparisons. In this table only the 19 genes were listed that showed a significant association in the STITCH network (see Figure 3) generated from the 8 hours isogenic comparison of *Salmonella* versus mock treated segments of pig 3. (_); non-significant ratio’s (<3 and >0.33) for above mentioned 19 genes extracted from micro array data files. In addition, ratios of differential expression measured in micro array experiments for all genes analyzed with QRT-PCR were listed (PCR). All genes showing a ratio of more than 3-fold up or down in both isogenic and inter-animal comparisons are presented in Additional file 1: Table S1.

### Isogenic micro array comparisons

**Figure 3:**
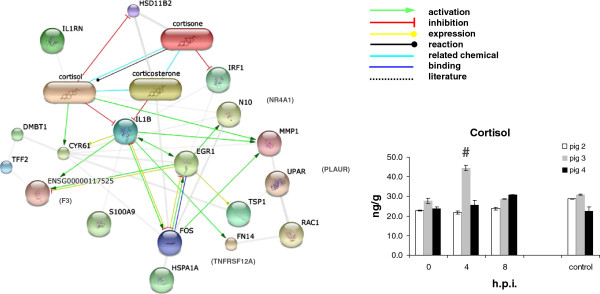
**Left panel: interactions between corticosteroids and proteins coding for genes differentially expressed in pig 3 8 h after challenge with *Salmonella*. Right panel (graph): cortisol levels in mucosal scraping of *Salmonella *treated (4 and 8 h) and mock treated (0 h and control) segments.** Bars represent the mean of 2 independent observations and error bars represent the variation between these observations. 0 hour bars represent the concentration of cortisol present in the intestinal segments before perfusion was applied (average concentration ± SD; 24.8 ng/g ± 2.6 [n = 6]). The bar marked with a number sign (#) was identified as a true outlier (i.e. was significant different) in a two-sided Grubbs’ test (*p* < 0.01) using the mean and standard deviation calculated over all determined values.

The commercial Operon array platform was used to analyze RNA extracted from segments according to the scheme depicted in Figure 1B and C. In Additional file 1: Table S1 ratios of all genes found differentially expressed for more than 3-fold up- or down are listed. The genes discussed in this manuscript are tagged with letter d and can be easily sorted from this table. In Table 1 the total number of genes regulated in these comparisons, and genes that responded to *Salmonella* in the 8 hour segments of pig 3 (isogenic comparisons; Figure 1B) were presented. In contrast to pig 2 and 4, in pig 3 a limited number of genes (37) were found regulated 8 hours after challenge with *Salmonella*, despite this pig showed a strong IL8 response similar as observed in pig 4. Moreover, compared to pig 4 (at 2 hours), and pig 2 (at 4 hours) this pig showed a relatively faint IL1B response at 2 hours that quickly descended to a normal level within 4 hours. Also, this pig failed to produce an REG3A response at 8 hours. Nevertheless, 21 out of the 37 genes regulated in pig 3 were similar to genes regulated in pig 2 and/or 4; e.g. the highly up-regulated CYR61, a gene that responded to several pathogenic bacteria in epithelial cells [19]. This suggested that exposure to Salmonella had proceeded equally well in all three pigs, and prompted us to investigate the role of the 16 genes exclusively regulated in pig 3 at 8 hours. Using bioinformatics program STITCH2 relevant protein-protein associations were detected for 19 out of the 37 genes, including associations of proteins/genes with corticosteroid metabolism (depicted in Figure 3 left panel and genes listed below TIMP1 in Table 1). Among these 19 genes was the enzyme hydroxysteroid 11-beta dehydrogenase B2 (HSD11B2), which interconverts cortisol and cortisone. Determination of cortisol in mucosal scrapings detected a significant higher level in pig 3 at 4 hours than in scrapings of pig 2 and 4 (Figure 3, right panel), suggesting that down-regulation of HSD11B2 at 8 hours in pig 3 was related to the metabolism of cortisol. In addition, a strong down-regulation of TFF2 and RAC1 mRNA expression was observed in pig 3 at 8 hours. TFF2 plays a role in mucosal protection and repair in the intestine and was found up-regulated at sites of ulceration in various chronic inflammatory diseases. The secreted *Salmonella*-effector protein SopE interacts with host Rho GTPases like RAC1 in epithelial cells and macrophages and stimulates RAC1-mediated cytokine production and cytoskeletal reorganizations, i.e. forming of membrane ruffles that facilitate invasion of these cells with *Salmonella*[20,21]. In pig 3, down-regulation of RAC1 could impair these processes and prevent or reduce the production of inflammatory cytokines like IL1B.

Sets of regulated genes in individual pigs were separately loaded into GeneDecks to assign genes to a specific pathway. Significant immunological pathways were selected from output files and presented in Additional file 2: Table S2 (sheet 8 h). The majority of pathways called for pig 2 and 4 reflected an inflammatory response induced by a bacterial infection, and were not called for pig 3. The genes of the MAPK and glucocorticoid receptor (GCR) signaling pathways regulated in pig 3 (down-regulation of RAC1 and up-regulation of HSPA1A, HSPA1B and NR4A1; see Table 1) showed little resemblance with the genes of these pathways regulated in pig 2 and 4. Together with the regulation of cortisol metabolism, the regulation of this set of unique genes in pig 3 may be part of “one” of the mechanisms by which the intestinal immune system tempers inflammation. However, cortisol-cortisone inter-conversion is a complex process. Therefore, further dedicated research is needed to observe similar responses in more than one pig to prove this hypothesis.

### Inter-animal micro array comparisons

The observed plasticity in response was used to detect “potential” genes involved in early induction/tempering of *Salmonella*-induced inflammation. Microarray comparisons between identical treated segments of the three different responding pigs (depicted in Figure 1C) detected a broad set of genes differentially expressed between these individual pigs at 2 and 4 hours. The genes showing a ratio of more than 3-fold up or down in inter-animal comparisons are presented in Additional file 1: Table S1 (sheets 2 and 4 h). IL8 and IL1B are important cytokines for the immune response in the intestine. Therefore differentially expressed genes of these inter-animal comparisons were grouped based on their similarity in response to that of IL8 at 2 hours and IL1B at 4 hours. From these mutual comparisons it was determined in which pig the highest level of expression was observed for a gene from our list. In case the highest expression of a gene was observed in pig 3 or 4 at 2 hours (the pigs that showed a strong IL8 response; see Figure 2) the gene was assigned to a group called IL8-high. For all other genes detected at 2 hours the highest expression was observed in pig 2 (the pig that showed no IL8 response; see Figure 2). These genes were assigned to a group IL8-low. A similar grouping was performed at 4 hours, except that assignment of genes was based on the IL1B response of pigs (IL1B-high; highest expression in pig 2 or 4, IL1B-low; highest expression in pig 3). In Additional file 1: Table S1 the assignment of genes to a list may be selected by sorting with the “list headers”.

### Pathway analysis inter-animal comparisons

Sets of regulated genes of the IL8-high and -low lists and the IL1B-high and -low lists at 2 and 4 hours were separately loaded into DAVID and GeneDecks to assign genes to a specific pathway. Significant immunological and chemical pathways involved in immunological processes were selected from output files and presented in Additional file 2: Table S2 (sheet 2 and 4 h). Genes specifically regulated in one list, but also part of a pathway called significant for the opposite list, are presented between brackets. Such genes may be important factors and may provoke an opposite/different effect. In contrast to the IL8-high list, no pathways were called involved in recognition of bacterial components by Toll-like receptors (TLR’s) and extracellular and cytosolic pattern recognition receptors (PRR’s) in the IL8-low list. These pathways are responsible for the first production of cytokines like IL1B (e.g. in inflammasomes [22]) or IL8. The appearance of two T-cell pathways in the IL8-low list, representing pig 2, which showed a delayed IL8 and IL1B response, suggests that T-cell activation of residing (e.g. intraepithelial lymphocytes [IELs] and/or γδ T cells) or infiltrated T-cells preceded the IL8 and/or IL1B response. The most prominent pathways called in the IL8-high list were the ERK signaling and Glucocorticoid Receptor Signaling pathway. Glucocorticoids (GC’s) are potent anti-inflammatory agents. ERK signaling in response to binding of ligands like macrophage migration inhibitory factor (MIF) to its receptor CD74 may abrogate the immunosuppressive action of GCR’s. NRF2-mediated Oxidative Stress Response was called in the IL8-high list. The influx of granulocytes (neutrophils, basophiles, and eosinophils) attracted by IL8 may be related to regulation of genes in this pathway. IL1B mediated inhibition of retinoic X receptor (RXR) function was called for both IL8 lists. However, a complete different set of genes in the high list was mapped to this pathway than for the low list. This pathway regulates the metabolism and transport of cholesterol and fatty acid/lipids, for which it is known that they modulate the immune response in the intestine. The list-specific regulation of genes in the PPAR signaling pathway (IL8-high) and of genes in the peroxisome/fatty acid oxidation pathway (IL8-low) confirms that differences in regulation of lipid metabolism are closely linked to regulation of inflammatory responses in the intestine.

In the IL1B-high list at 4 hours regulation of Glucocorticoid Receptor and PPAR signaling pathway-genes continued. In the IL1B-low list, representing the pig [3] in which the IL1B and IL8 response decreased to normal levels, and in which the inflammatory response to *Salmonella* was tempered after 8 hours, two lipid associated pathways, i.e. the synthesis and degradation of ketone bodies (by-products of fatty acids metabolism) and the ”Arachidonic acid metabolism”, were called. In the opposite IL1B-high list, several different genes mapped to these pathways were regulated; suggesting lipid metabolism and Arachidonic acid related immune modulators like prostaglandins are involved in regulation of *Salmonella*-induced inflammation. Calling of the “Agranulocyte and Granulocyte Adhesion and Diapedesis” pathways at 4 hours indicates that activation and/or an influx of lymphocytes and monocytes from the blood to the epithelial layer occurred in pigs of the high-ILB list. Probably also related to this influx/activation was the mapping of genes to the “Phagosome” and “Antigen processing and presentation” pathways. Both pathways are involved in engulfing of bacteria and processing them to exposable antigen fragments. The inflammatory state in the two pigs of the IL1B-high list is probably best illustrated by mapping of genes to the “Acute Phase Response Signaling” and “p53 signaling” pathway. Genes in the latter pathway point to a response to damaged DNA, and to induction of apoptosis.

Completely different genes of the “ErbB signaling” pathway were regulated in the IL1B-low and -high lists. High list genes AREG-NRG1 induces AP1-mediated (JUN) transcription, and low list genes NCK1 and GAB1 are involved in cytoskeleton rearrangements in intestinal epithelial cells in response to bacterial adhesion or invasion. Also completely different ring-finger type E3 ubiquitin ligase complex-genes were regulated in the low and high list, suggesting that forming of different type of ubiquitination complexes results in degradation of different proteins. For instance, genes in the low list are part of the SCF Ubiquitin ligase E3 complex (BTRC-RBX1) that ubiquitinates NFKBIA and PER1. The latter protein affects hypoxia-responsive element mediated transcription.

### Transcription networks and selection of key factors and substances

**Figure 4:**
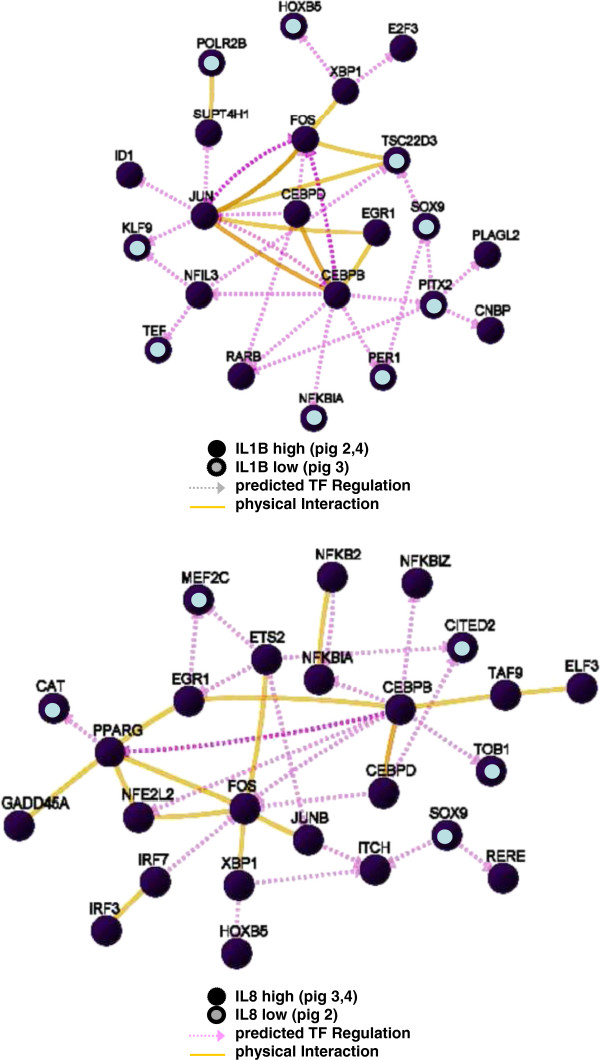
**Interaction between transcription factors/regulators found differentially expressed in inter-animal microarray comparisons of *Salmonella *treated loops at 2 hours (bottom) and 4 hours (top).** TF: transcription factor.

A graphical network was built of all transcription factors/regulators present in gene-lists of both 2 and 4 hours inter-animal comparisons. Genes which showed an interaction to at least one other gene of the sub-list were displayed and non-interacting genes were omitted from the network (Figure 4). Genes central in these networks were selected as key factors, and a comprehensive data-mining was performed to find associations with chemical substances that have potential to influence the transcription of these genes, and/or the cellular processes regulated by these genes (see Methods). In Table 2 selected chemical substances tested, together with their corresponding “central” key factor genes, are listed. In addition, the genes HSD11B2, F3, NR4A1, and MMP1, which according to our STITCH network (Figure 3) may play a role in the cortisol/cortisone regulated tempering of inflammation in pig 3 at 8 hours, were also selected as key factor genes. Preferably, chemical substances were selected, which according to literature had the potential to influence expression of more than one key factor (maximal 3 key factor genes per chemical were listed in Table 2).

**Table 2:** **Percent inhibition or stimulation of *Salmonella*-induced IL8 and NFKBIA mRNA expression**

**Chemical**	**Key factor genes (3 listed)**	**CAS # chemical group**		**NFKBIA**		**IL8**	
			**μM**	**%**	**%**	**%**	**%**
			hours	4	20	4	20
Genistein	FOS	446-72-0	0.5	80	57	18	54
	CEBPB & D	Isoflavones	5	71	56	12	66
	PITX2		50	53	27	3	11
Quercetin	IL1B	117-39-5	2	92	61	86	90
	NFKBIA	Flavonols	20	111	75	86	92
	F3		200	241	52	19	26
Indomethacin	EGR1	53-86-1	1	106	66	86	93
	NR4A1	Indoles	10	123	129	86	101
	NFKBIA		100	261	108	19	42
			hours	3	6	3	6
Curcumin	ETS2	458-37-7	1	186	111	275	138
	CEBPB	Diarylheptanoids	10	412	100	843	37
	NFE2L2		100	27	10	86	24
Chenodeoxycholic Acid	PPARG	474-25-9	1	217	145	291	317
	NR4A1	Bile Acids and Salts	10	407	140	468	293
	XBP1		100	591	211	779	318
Nordihydroguaiaretic Acid	HSD11B2	500-38-9	1	226	118	141	85
	FOS	Lignans	10	222	78	214	123
	IL8		100	15	6	2	1
(E)-Guggulsterone	NFKBIA	6439929	1	135	45	77	308
	NFE2L2	Plant Gums	5	96	32	69	139
	STAT3		50	21	8	21	13
(Z)-Guggulsterone	NFKBIA	6450278	1	75	43	23	176
	NFE2L2	Plant Gums	5	46	35	18	122
	STAT3		50	47	16	10	4
Sodium Selenite	JUNB	10102-18-8	10	83	31	96	44
	FOS	Selenium Compounds	100	100	37	115	37
	PITX2		1000	143	53	30	12

IL8 and NFKBIA mRNA expression was measured with QRT-PCR in IPEC-J2 cells in the absence or presence of different concentration of chemicals (μM), after 4 and 20 hours (top panel) or after 3 and 6 hours (bottom panel) of incubation. Chemicals were selected based on their association with the in this study identified key transcription factor/regulators genes (maximal 3 genes were listed per chemical). For each chemical CAS numbers (#) and the group of chemicals to which they belong were listed.

### Effect of selected chemicals on *Salmonella*-induced inflammation in IPEC-J2 cells

**Figure 5:**
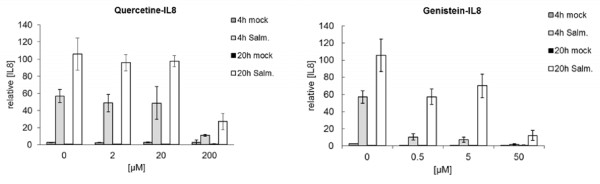
**Relative concentration of IL8 mRNA measured by QRT-PCR in *Salmonella *and mock treated IPEC-J2 cells after 4 and 20 hours in the absence (0 μM bars) and presence of different concentrations of Quercetine (left graph) or Genistein (right graph).** Bars represent the mean of 2 independent observations. Error bars represent the variation between these 2 independent observations.

To perform a first evaluation whether the 20 selected chemicals have potential to influence *Salmonella*-induced gene expression in IPEC-J2 cells, we used IL8 and NFKBIA as reporter genes. The expression level of both these mRNA’s was found up-regulated in IPEC-J2 cells (see Figure 5; μM bars *Salmonella* versus mock) and in SISP loops (see Table 1) after challenge with *Salmonella*. All chemical were tested for 4 and 20 hours or for 3 and 6 hours incubation periods at three different concentrations chosen around a concentration that affected expression of the genes/proteins in question in cultured cells (thus, in the absence of *Salmonella*) in earlier studies (studies linked to data in the Comparative Toxicogenomics Database: see Methods). For all chemicals, the turbidity of the culture medium was increased after 6 or 20 hours, even at the highest concentration of chemical tested, indicating that the chemicals did not seriously affected the growth of Salmonella. In Figure 5 an example of the concentration dependent regulation of IL8 mRNA expression by Quercetin and Genistein is depicted for *Salmonella* and mock challenged wells. In case IPEC-J2 cells were not challenged with *Salmonella*, none of the 20 chemicals stimulated or inhibited the expression of IL8 and NFKBIA (results not shown). For *Salmonella*-challenged wells the stimulation/ inhibition index was calculated from the relative concentrations IL8 and NFKBIA mRNA measured in the presence of different concentration chemicals at the incubation times applied (see Methods). For 10 of the chemicals related to the key transcription factors/regulators (listed between brackets below) no significant stimulation/inhibition of *Salmonella*-induced IL8 or NFKBIA gene expression was observed (Palm oil [NFIL3], alpha-Tocopherol [PPARG], Tretinoin [PER1, TEF], Zymosan [NFKBIA], Aminolevulinic Acid [MMP1], beta-Carotene [CAT], Butyric Acid [IL8], Lycopene [NFKBIA], and Folic Acid [ETS2]). Concentrations of 10 μM or more Menadion (alias; Resveratrol [GADD45A]) resulted in the loss of IPEC-J2 cells from the monolayer already after 3 hours. The observed effect of Menadion on expression of both reporter genes was, therefore, doubtful (results not shown). For the 9 chemicals that significantly affected Salmonella-induced IL8 and NFKBIA mRNA expression the calculated inhibition/stimulation indexes were summarized in Table 2 and bar plots are presented in Additional file 3: Figure S1. Except for Chenodeoxycholic Acid, for which a consistent stimulation of both NFKBIA and IL8 mRNA was observed, most chemicals inhibited mRNA expression of IL8 and/or NFKBIA. However, stimulation and inhibition was observed for Nordihydroguaiaretic acid and Curcumin, depending on the concentration tested.

## Discussion

The main goal of this study was detection of “first-response” genes that have a major impact on development of *Salmonella*-induced inflammation latter on. To avoid repetition with studies in which the transcriptional response to *Salmonella* in the intestine of rats [23,24], mouse [25], chicken [26,27], and pigs [9,28,29] was recorded after longer infection periods than 2 and 4 hours, the pathways/genes called significant after 8 hours were discussed briefly in the results section (section isogenic comparisons). In this discussion we focus on 2 and 4 hours genes/processes which may play a crucial role in the regulation of inflammation in the intestine in general.

One of the most important observations in this study was the failure of pig 3 to produce an ongoing IL1B response even though this pig produced a faint IL1B and high IL8 response at 2 hours. Already after 2 hours of perfusion we detected invasion of *Salmonella* in all 3 test pigs [9]. It is known that crossing of *Salmonella* over the epithelial barrier is also supported by Microfold (M) cells. Research in humans and mouse revealed that M cells are enterocyte-like cells formed in the Peyers’s patches of the jejunums and ileum. These cells lack microvilli and are able to phagocytize pathogenic organisms/particles and transport them over the epithelial barrier into the lamina propia [1]. After these cells become injected with *Salmonella* effector proteins [6] or invaded with whole *Salmonella* bacteria, M cells undergo cytoskeletal rearrangements to support forming of *Salmonella* containing vacuoles [19] and produce an array of cytokines, among them IL8, IL1B and macrophage inflammatory proteins (MIP’s) [2]. IL8 and MIP’s attract and activate neutrophils, basophils, monocytes (macrophages and DC’s), and T-cells. If M cells in pigs are also capable to produce a similar cytokine response to *Salmonella*, attraction of these cells may have occurred in pig 2 and 4, and stayed behind in pig 3, and with this, also inflammation induced by these cells. In analogy, we observed a response of IL1B and MIP’s mRNA (CXCL2, 5), and CXCL6 (alias; granulocyte chemotactic protein 2) at 4 and 8 hours in pig 2 and 4, but not in pig 3. However, it had to be noted that “normal” enterocytes and residing immune cells can also account for this “first” IL8 and IL1B response, respectively.

Using the “tissue expression” module of DAVID equal proportions of IL8-high and -low genes (17 and 15%, respectively) were mapped to a BDCA4 + DCs expression library (result not shown) indicating that activation of DC’s also occurred in the absence of an IL8 response. Whether this are residing or infiltrating DC’s is not clear. We detected a significant number of genes part of the TSLP Signaling pathway in IL8-high pigs (CISH, GAB1, IL8, MAP2K1, NFKB2, NFKBIA) at 2 hours, a pathway responsible for cross-talk between enterocyte-conditioned DC’s (EC-DC’s) and enterocytes [2]. TSLP mediated cross-talk directs T cell polarization towards a non-inflammatory T helper type 2 (Th2) response [30], and over production of TSLP results in an exaggerated basophil responses, believed to be responsible for induction of Th2 cytokine-associated inflammatory diseases like asthma and food allergy [31]. However, all TSLP-pathway genes we detected are also essential factors in many other immunological signaling systems. Therefore, we cannot conclude whether TSLP-crosstalk, and with this T cell polarization important for development of inflammation, was differently regulated in the IL8-low pig and two high pigs.

Neutrophils are the first and predominant cells that invade the intestinal site of *Salmonella* infection [32,33]. Besides neutrophils engulf pathogenic bacteria, degrade them in fused phago-lysosomes, and express degraded material as MHC class II on their surface, they also produce chemokine’s like the above mentions IL8, CXCL5, and CXCL6. Moreover, neutrophils express cytokines that activate (TNF-α), or suppress (IL10 and TGFβ) T cell responses, regulate Th1/Th2 polarization (IL4 and IL12, respectively), and supports Th2-Th17 differentiation (IL6) [34]. These functions make neutrophils important orchestrators of the first (innate) response in the intestine to bacterial pathogens. In relation to this, IL8-high genes mapped to the “NRF2-mediated Oxidative Stress Response” pathway may support the destruction of *Salmonella* in phago-lysosomes of neutrophils. The gene SCARB1, mapped to this pathway, was higher expressed in the IL8-low pig 2. SCARB1 is a scavenger receptor located in lipid rafts that facilitates the flux of free and esterified cholesterol between the cell surface and extracellular donors such as high-density lipoproteins (HDL’s). Through its phosphatidylserine-binding activity SCARB1 also plays a role in phagocytosis. Recently it was shown that HDL’s were potent attenuators of neutrophil activity and that free cholesterol alters neutrophil lipid raft structure, and consequently, Ca^2+^ entry and respiratory burst in these cells [35]. Therefore, SCARB1-mediated cholesterol efflux may influence the activity of neutrophils. In addition, the internal cholesterol load in these phagocytes may be regulated by SCARB1, affecting cortisol metabolism and with this the inflammatory status of the intestine. Besides SCARB1 several other genes, directly or indirectly involved in conversion, transport, or regulation of steroids, were differently expressed in the IL8-low and high pig(s) (e.g. HSD17B6, HMGCS1, SC4MOL, CYP2C8, 9 and 18). The most interesting question to be answered now is which type of phagocytic cell plays a dominant role in early regulation of steroid metabolism.

Two pigs (3,4) that produced an early IL8 and IL1B response at 2 hours activated TLR, RIG1, cytosolic DNA-sensing, and/or NOD signaling pathways responsible for sensing of bacterial “danger signals”. In contrast, in pig 2, in which the IL8 and IL1B response was delayed, no pattern-recognition pathways were called significant. Remarkably, regulation of genes coding for T-cell receptor delta (δ; TRD) and gamma (γ; CD3G) chains was observed in this pig, suggesting that γδ T cells were active at the site of exposure to *Salmonella*. γδ T cells are the predominant T cells within the population of intraepithelial lymphocytes (IELs) in the intestinal mucosa. They play an early and essential role in sensing ‘danger’ by invading pathogens. In case of an acute *Salmonella* infection their concentration expands dramatically [36]. γδ T cells do not require presentation of antigens by the MHC-complex and are believed to have a prominent role in recognition of lipid antigens [37]. We observed a different expression of mRNA’s coding for enzymes supporting the oxidation of fatty acids in the peroxisome in IL8-low pig 2 than in the two other pigs. Among these enzymes ACSL3, a key enzyme in the β-oxidation of unsaturated fatty acid, which transcription is regulated by the PPARG - retinoid X receptor (RXR) signaling. It was reported that IL1B-mediated inflammation targets the RXR-alpha receptor (RXRA) for nuclear export and degradation [38]. In addition, our analysis showed that a complete different set of genes belonging to the “LPS/IL-1 mediated inhibition of RXR function” pathway was regulated in the IL8-low pig than in the two high pigs. This suggests that inhibition of PPARG-RXRA signaling influences gene expression of enzymes responsible for β-oxidation of unsaturated fatty acid, and with this peroxisome-mediated processing of lipid antigens recognized by γδ T cells. It would be interesting to investigate by which cells peroxisome processing and presentation of such bacterial lipid antigens is facilitated, M cells or other antigen presenting cells (APC’s), and whether γδ T cells play a major role in recognition of these lipids antigens.

Differences in the level of expression of specific genes between IL8-high and -low pig(s), which may be important for T cell proliferation, survival and activation, were observed. In the IL8-low pig, transducer of ERBB2-1 (TOB1) and MEF2C were expressed higher, and in the high pig NR4A1, FOS, CISH, and NFKBIA. TOB1 associates with SMAD4 and exerts an inhibitory effect on IL2 transcription in T cells [39-41]. IL2 expression by antigen-activated T cells plays a critical role for orchestrating the immune response. It stimulates the proliferation of T and B cells, NK cells, and cells of the monocyte lineage (macrophages, DC’s). In normal T cells, triggering of the TCR-CD3 complex leads to the activation of transcription factors involved in immune processes, including the here detected genes NFKB2, EGR1, and FOS. MEF2C is necessary for the transcriptional activation of IL2 and plays a crucial role in T-cell apoptosis by regulating expression of NR4A1 [42]. As shown in Figure 3, EGR1, FOS, and especially NR4A1, a potent regulator of transcription of steroid enzymes [43], all play a role in tempering inflammation in pig 3. Transcriptional regulation by TOB1 and MEF2C in the IL8-low pig may therefore be crucial for the function of IELs and with this the production of cytokines that orchestrate communication between the various immune cells in the intestine early after exposure to *Salmonella*.

Glucocorticoid Receptor (NR3C1) and ERK signaling were the most prominent pathways called in the IL8-high pigs. The GCR-GC complex associates with CEBPA, resulting in CDKN1A (alias p21) production and induction of cell-cycle arrest. CDKN1A was higher expressed in ILB-high pigs 2 and 4 at 4 hours. In addition to *Salmonella* effector protein AvrA [44], GC’s represses NFKB-driven production of interleukins and other inflammatory cytokines mediated by transcription factor NFKB in APC’s (macrophages, DC’s) and granulocytes. GC’s specifically stimulate the expression of NFKB inhibitor NFKBIA [45]. Interestingly, ANGPT2, part of both these pathways, was expressed to a higher level in the IL8 low pig 2. ANGPT2 (angiopoietin 2) binds to the same receptor (angiotensin II receptor type 1) as angiotensin II. Activation of this receptor leads to nuclear translocation of ERK1/2 resulting in activation of transcription factors we detected in this study, like FOS, CITED2, NFIL3, PER1 (NFIL3 and PER1 are discussed below), and member of the ETS family of transcription factors (see IL8-low list: ETS2, ELF3). Angiotensin II is a potent activator of cortisol by stimulating NR4A1 binding to its transcriptional response element [46].

The level of Bactericidal/permeability-increasing protein (BPI) mRNA in IL1B-low pig 3 was higher than in pig 2, the pig with a delayed IL8 and IL1B response and that responded most vigorous to *Salmonella*. BPI possesses antibacterial, endotoxin-neutralizing and opsonic activity against Gram-negative bacteria, among them *Salmonella*[47]. Expression of BPI was detected in mucosal epithelia and neutrophils. Recently it was shown that all-trans retinoic acid promotes binding of CEBPB (up-regulated in case an elevated IL8 and/or IL1B expression was observed; see Additional file 1: Table S1) or CEBPE to the BPI promoter and stimulates BPI expression in human myeloid cells [48]. After normalization of IL1B levels in pig 3 at 4 hours degradation of RXRA may stop (see above) and signaling through this receptor may be restored, and with this CEBPB-driven BPI expression. With respect to BPI, it would be interesting to investigate whether the expression level of this bactericidal correlates with colonization and survival of *Salmonella* bacteria in the GI tract of pigs under natural conditions.

In the IL1B low pig three transcription factors/regulators, PER1, NFIL3 (nuclear factor regulated by IL-3) and TEF, were mapped to the circadian rhythm pathway. TEF and NFIL3 compete for the same “PAR DNA”-transcriptional binding site and both play an important role in transcriptional regulation from the interleukin-3 promoter [49-51] and with this, in IL3-mediated production and differentiation of granulocytes and monocytes (macrophages and DC’s). In addition, NFIL3 represses PER1 transcription [52], a factor that affects transcription from the “Hypoxia-Responsive Element” [53]. Also, transcription of four other transcription factors/regulators from our gene lists (ALAS1, EGR1, IRF1 and DBP) is regulated by the circadian rhythm system. NFIL3 is highly expressed in CD14+ monocytes (**BioGPS**), suggesting that transcriptional regulation by TEF, NFIL3 and PER1 could affect the respiratory burst-mediated destruction of *Salmonella* in the phagozomes of macrophages.

We observed an effect on mRNA expression of *Salmonella*-response genes IL8 and NFKBIA in IPEC-J2 cells for 9 out of the 20 chemicals. It has to be noted that we tested pure chemicals in a “clean, *in vitro* environment and in one type of intestinal cells. *In vivo*, these substances are part of a complex matrix (food-feed) and are subjected to modifications induced by other chemicals, host enzymes, and micro biota during their route from intake to the intestine. Therefore, further *in vivo* studies, in which all types of functional intestinal cells and immune cells are exposed to pre-digested food or feed preparations, have to prove if these chemicals of natural origin indeed influence inflammatory processes in the intestine of pigs. Moreover, in such experiments the influence of these chemicals on *Salmonella* colonization and invasion of the intestinal mucosa of the pig may be studied under natural conditions.

## Conclusions

We describe a set of key transcription factors/regulators that may be used as molecular tools to further elucidate the “very early” immune mechanisms decisive for the state of inflammation of the intestine later on. Better insight in these processes may lead to the development of new food/feed additives that are capable to steer inflammation in the intestine.

## Competing interests

The authors declare that they have no competing interests.

## Authors’ contributions

SV carried out micro-array experiments. AdW carried out QRT-PCR and IPEC-J2 in vitro assay’s, JvdM designed, planned and performed SISP experiments. All authors read and approved the final manuscript.

## Supplementary Material

Additional file 1: Table S1 Title of data: Differential expressed probes of microarray experiments / STITCH associations. Description of data: Interactive table of ratios of differential expressed probes (Sheet of 8 hours isogenic, and sheet of 2 and 4 hours inter-animal comparisons)/Table with type and confidence scores of associations between genes/proteins and chemicals of STITCH analysis. Click here for file

Additional file 2: Table S2 Title of data: Pathway analysis of genes differentially expressed. Description of data: Table of significantly called pathways of gene-lists of 2 and 4 hours inter-animal and 8 hours isogenic comparisons. Click here for file

Additional file 3: Figure S1 Title of data: Inhibition or stimulation of Salmonella-induced IL8 and NFKBIA mRNA in IPECJ2 cells by chemicals. Description of data: Bar plots of QRT-PCR measurements of IL8 and NFKBIA mRNA expression in IPEC-J2 cells. Click here for file
