# Supplementary material for: Transcription networks responsible for early regulation of Salmonella-induced inflammation in the jejunum of pigs
Source: J Inflamm (Lond). 2013 Apr 17;10:18. doi: 10.1186/1476-9255-10-18 (PMC3637394; doi:10.1186/1476-9255-10-18)

Supplementary Fig. S1; : Inhibition or stimulation of *Salmonella* -induced IL8 and NFKBIA mRNA in IPECJ2 cells by chemicals.

top panel (3 and 6 hours)

Relative concentration of IL8 and NFKBIA mRNA (Y-axis) measured by QRT-PCR in Salmonella (+Salm) and mock (-Salm) treated IPEC-J2 cells after 3 and 6 hours, in the absence (0 µM bars) and presence of different concentrations of chemical (µM bars). For each concentration of chemical two independent observations are presented.

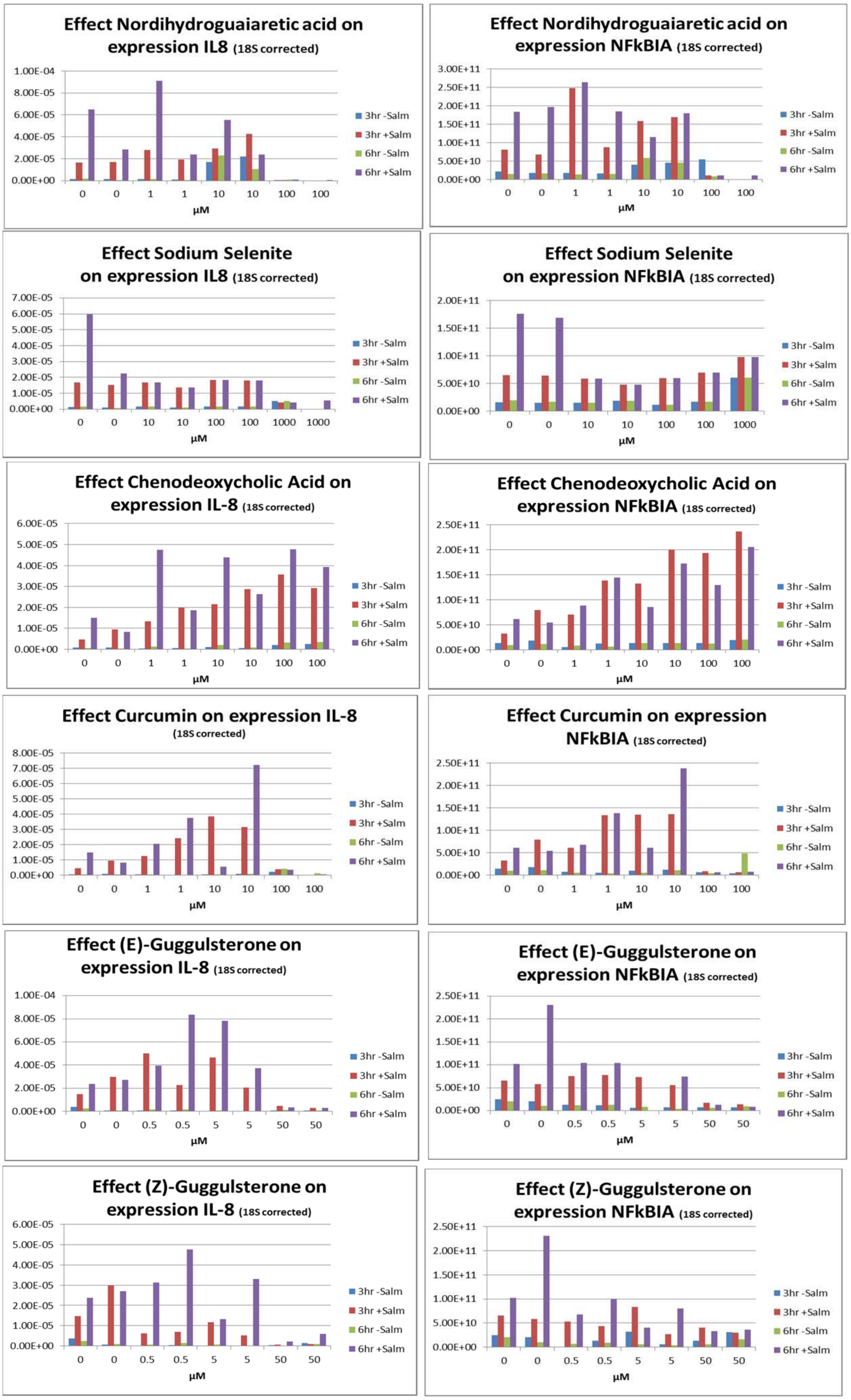

bottom panel (4 and 20 hours)

Relative concentration of IL8 and NFKBIA mRNA measured by QRT-PCR in Salmonella and mock treated IPEC-J2 cells after 4 and 20 hours, in the absence (0 µM bars) and in the presence of different concentrations of chemical (µM bars). Bars represent the mean of 2 independent observations. Error bars represent the variation between these 2 independent observations.

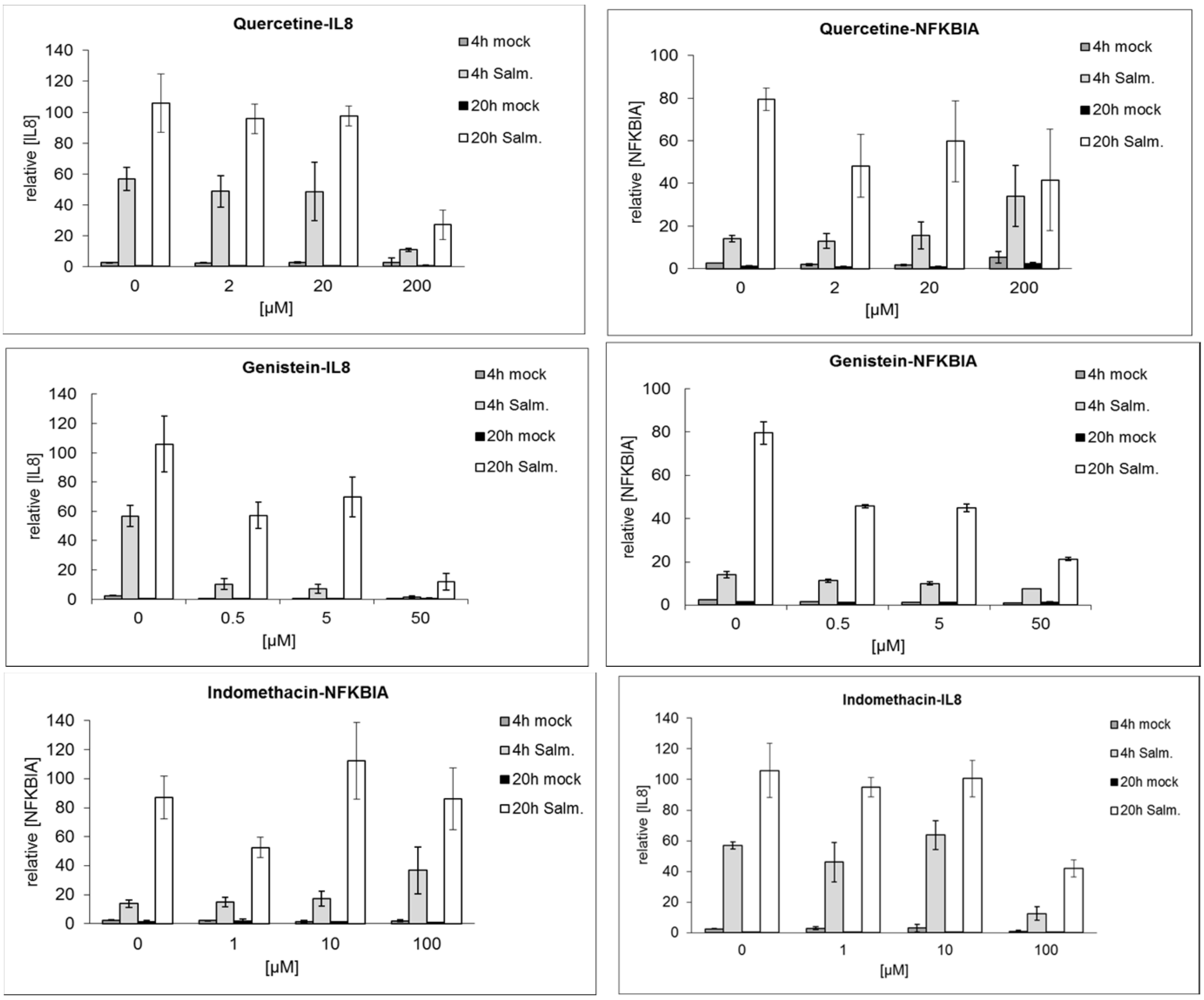

Supplement: Additional file 3: Figure S1 — Title of data: Inhibition or stimulation of Salmonella-induced IL8 and NFKBIA mRNA in IPECJ2 cells by chemicals. Description of data: Bar plots of QRT-PCR measurements of IL8 and NFKBIA mRNA expression in IPEC-J2 cells. [file 1476-9255-10-18-S3.pdf]
